# VALIDITY OF THE EKBLOM-BAK CYCLE ERGOMETER TEST IN PATIENTS WITH CARDIOVASCULAR DISEASE

**DOI:** 10.2340/jrm.v56.39901

**Published:** 2024-08-13

**Authors:** Magnus FRANSPLASS STORLI, Marius STEIRO FIMLAND, Harald Kåre ENGAN, Jon Arne SANDMÆL

**Affiliations:** 1 Department of Neuromedicine and Movement Science, Faculty of Medicine and Health Sciences, NTNU Norwegian University of Science and Technology, Trondheim; 2 Unicare Helsefort Rehabilitation Centre, Rissa; 3 Unicare Norway, Oslo; 4 Unicare Røros, Røros, Norway

**Keywords:** cardiovascular disease, submaximal test, cardiorespiratory fitness, rehabilitation

## Abstract

**Objective:**

To assess the validity of the Ekblom-Bak cycle ergometer test in patients with cardiovascular disease admitted to cardiac rehabilitation.

**Methods:**

Estimated peak oxygen consumption from the Ekblom-Bak test was compared with directly measured peak oxygen consumption from a treadmill cardiopulmonary exercise test. Patients completed the cardiopulmonary exercise test first, followed by the Ekblom-Bak test after 24 h rest. Pearson’s correlation coefficient (r) was used to establish the correlation between estimated and measured peak oxygen consumption, and Bland–Altman plots with limits of agreement were used to determine the bias between the 2 tests.

**Results:**

Twenty-six patients were included in the final analysis. The Ekblom-Bak test significantly overestimated peak oxygen consumption. Agreement between estimated and measured peak oxygen consumption was: bias = 4.3 mL/kg/min (limits of agreement: –4.0–12.6 mL/kg/min).

**Conclusion:**

The Ekblom-Bak test overestimated peak oxygen consumption to such an extent that it cannot accurately assess cardiorespiratory fitness in patients with cardiovascular disease. Thus, the cardiopulmonary exercise test remains the test of choice.

## LAY ABSTRACT

Measurement of cardiorespiratory fitness is highly recommended both before and after cardiac rehabilitation, to optimise exercise programmes and to measure the effect of the rehabilitation. A cardiopulmonary exercise test is the gold standard exercise test. However, this is an expensive and time-consuming method. Thus, we wanted to investigate whether the relatively new Ekblom-Bak cycle ergometer test, which is cheaper and more easily administered, would be able to estimate cardiorespiratory fitness accurately in patients with cardiovascular diseases admitted to cardiac rehabilitation. Based on the findings in this study, the Ekblom-Bak cycle ergometer test does not seem to be able to measure cardiorespiratory fitness accurately in patients with cardiovascular diseases. Therefore, the cardiopulmonary exercise test remains the test of choice.

Cardiovascular diseases (CVD) are a major cause of disability worldwide (1, 2). Admission to cardiac rehabilitation has been shown to improve exercise capacity, cardiovascular risk factors, quality of life, and mortality rates and is thus highly recommended for patients with CVD (3–5). Assessment of cardiorespiratory fitness (CRF) by use of exercise testing is recommended both before and after cardiac rehabilitation, as it is a strong predictor of physical performance, health outcomes, and longevity (6–8). The cardiopulmonary exercise test (CPET) is the gold standard exercise test for direct measurement of CRF and enables risk stratification for several CVD diagnoses (9–11). In addition, it provides a framework for determining suitable aerobic exercise intensity, making it a useful clinical tool recommended by the American Heart Association (12, 13).

However, CPET requires maximal exercise effort, which is not feasible for certain patients with CVD due to symptoms or psychological barriers (9, 14). Patients with CVD may experience symptoms such as unstable angina, arrhythmias, palpitations, dizziness, and dyspnoea, in addition to psychological barriers related to the possibility of acute cardiac events (9, 14–16). Furthermore, proper interpretation of CPET results is dependent on physiological expertise, and it requires the use of expensive equipment for analysing expired air in a controlled lab environment (17).

When a CPET is not feasible in a clinical setting, submaximal exercise tests can be used to predict CRF. The Åstrand test is one of the most commonly used cycle ergometer tests and predicts CRF by extrapolating steady-state heart rate (HR) achieved after 6 min at an individually chosen work rate (18, 19). It is relatively easy to administer and commonly used in fitness evaluations and as a substitute for CPET in some studies (20–23). The Ekblom-Bak Cycle Ergometer Test (Ekblom-Bak test) is a newer submaximal exercise test that estimates CRF with higher precision in healthy adults than the Åstrand test (24–26). The Ekblom-Bak test equations were updated by Björkman and colleagues in 2016, and CRF estimations are calculated from HR response between 2 work rates, rather than the single work rate used in the Åstrand test. The revised version is considered to provide valid estimates of CRF for a wide variety of both age and fitness levels within the healthy adult population (24).

While the Ekblom-Bak test is easily administered, time-efficient, and carries relatively low risk, evidence regarding its validity to estimate CRF in various patient populations is scarce. There is currently no evidence of the Ekblom-Bak test’s accuracy in estimating CRF for patients with CVD, for which some use beta-blockers known to lower HR, making its applicability in cardiac rehabilitation settings uncertain. Thus, the aim of this study was to assess the validity of the Ekblom-Bak test in patients with CVD admitted to cardiac rehabilitation.

## METHODS

### Patients

Patients were recruited from an inpatient cardiac rehabilitation programme (Unicare Røros, Norway). The patients were referred for rehabilitation by their general practitioner or the responsible physician at the hospital. They self-reported categorical answer mode questionnaires as part of their rehabilitation, which included demographics, physical function, quality of life, and anxiety and depression. Information on disease history, diagnosis, and medication was collected from the patient journals. A convenience sample approach was used, including every patient willing to participate and medically cleared by the cardiologist at the centre. Patients were given oral and written information and provided written consent. Data were collected in accordance with the requirements of the Declaration of Helsinki, and the study was approved by the Norwegian Regional Ethical Committee South-East B (Project ID: 281364).

### Test information

The CPET was part of the clinical routine of the cardiac rehabilitation, and every patient performed the test during the first 3 days of their stay. Before both the CPET and the Ekblom-Bak test, patients were instructed to abstain from food intake, nicotine, and consuming fluids other than water for the last 2 h before the test, as well as not performing any vigorous physical activity during the last 24 h prior to the test. To accommodate this, the test schedule was designed so that the Ekblom-Bak test was performed 24–26 h after the CPET. One test leader administered all the CPETs, and a second test leader administered all Ekblom-Bak tests. Instructions on Borg’s scale were given prior to both tests, as the present clinical routines of the CPET and the specific protocol of the Ekblom-Bak test operated with different versions. The Borg Category Ratio 10 scale (Borg CR10) ranging from 0–10+, and Borg’s Rating of Perceived Exertion (Borg RPE) ranging from 6–20, were utilized for the CPET and Ekblom-Bak test, respectively (27). The patient’s weight was registered to the nearest 0.1 kg prior to both tests using an electronic weight (Tanita BWB-800, Tokyo, Japan).

### Cardiopulmonary exercise test

Patients performed a maximal incremental walking protocol on a treadmill (Woodway PPS 55 Med, Waukesha, WI, USA). During the test, patients were fitted with a face mask of appropriate size (Hans Rudolph, Shawnee, KS, USA), connected to an inspiratory flow meter that linked to the Vyntus CPX (Vyaire medical, Hoechberg, Germany). Vyntus CPX measured oxygen (O_2_) consumption through breath-by-breath analysation of the gas exchange between carbon dioxide (CO_2_) and O_2_. The volume of the inspiratory flow meter and gas were calibrated by the Vyntus CPX’s automatic procedures prior to each test, using a calibration gas (5.00 ± 0.01% CO_2_ and 16.00 ± 0.01% O_2_, (Vyaire Medical, Hoechberg, Germany) and ambient indoor air. Prior to the test, patients were instructed on test procedures and Borg CR10, and their height and waist circumference were measured to the nearest cm. Patients were connected to a 12-lead electrocardiogram (ECG) recording (Custo cardio 300BT_A, Ottobrunn, Germany) and a blood pressure measurement device with the cuff placed on the upper right arm (Tango M2, SunTech Medical, Morrisville, NC, USA). Resting ECG and resting blood pressure were recorded from a seated position before the CPET was initiated. ECG readings were continuously monitored by a cardiologist throughout the test, while blood pressure was measured every 3 min, and at the cessation of the test. Concurrently with blood pressure measurements, patients were asked to report their subjective feeling of exertion according to BORG CR10.

Testing was performed using specifically developed walking protocols for patients with CVD, which were created by a hospital specializing in treatment and rehabilitation of cardiac patients (Feiringklinikken, Feiring, Norway). Four ramping protocols was programmed in the Sentrysuite test software (Vyaire Medical, Hoechberg, Germany), and increments were automatically controlled by the software. Protocols differed in both initial (1.6–3.5 km/h), and final (4.0–6.0 km/h) walking velocity, with maximal velocity occurring after ~9 min. Every protocol started at 0% inclination, with a slow gradual increase to 4% inclination during the first ~9 min. This was followed by larger increases for the remainder of the test, with increments up to a maximum of 20% inclination occurring after ~15 min. The protocol was chosen by the test administrator, based on the patient’s self-reported physical activity level over the last couple of months, history of disease, severity of and time since cardiac event, and observation of gait pattern. No warm-up was performed prior to testing, as the slow ramping during the first minutes of the protocols served this purpose. Patients were instructed to avoid holding on to the handrails if not absolutely necessary throughout the entire test, and to relax their arm during blood pressure measurements to ensure optimal recordings. If a patient seemed to have been assigned a suboptimal protocol, the workload was manually adjusted to ensure that maximal exhaustion was obtained. Tests were terminated when patients reached voluntary exhaustion in the form of, e.g., dyspnoea or leg fatigue, or if any of the indications for test termination listed by the American Heart Association were observed (28). Test results are expressed as peak O_2_ consumption (V̇O_2_peak). V̇O_2_peak is a commonly used measurement of CRF in patients with CVD, and expresses the highest O_2_ consumption during exercise to voluntary exhaustion (29). No cut-off value was set for the respiratory exchange ratio (RER). Absolute V̇O_2_peak (L/min) was calculated from the relative V̇O_2_peak (mL/kg/min) value reported by the Sentrysuite test software, using the formula: V̇O_2_peak (L/min) = ((V̇O_2_peak (mL/kg/min) × weight))/1,000.

### Ekblom-Bak test

Test procedures for the Ekblom-Bak test are described in the article concerning where the test was created (25), and the full protocol can be found at the homepage of the Swedish School of Sport and Health Sciences (https://www.gih.se/ekblombaktest). The test was performed on an electronically braked cycle ergometer (Monark model 928E, Vansbro, Sweden), while patients wore an HR monitor on the chest (Polar model H7, Kempele, Finland). The test administrator ensured that the patient had complied with the pre-test criteria and provided instructions on test procedures and Borg RPE. Patients were instructed to pedal with a cadence of 60 revolutions per min. Total test duration was ~8 min, with the first 4 min being performed at a fixed work rate of 30 watts (W). This was directly followed by 4 min at a higher predetermined individualized work rate. The test leader chose the higher work rate based on clinical judgement, considering factors such as gender, age, training status, and information on disease, aiming to achieve a work rate corresponding to Borg RPE ≈14. After the first minute of the higher work rate, patients were asked to assess their current Borg RPE. If Borg RPE was less than 12, the work rate was further increased by 30 W, and the final 4-min period was restarted. If Borg RPE was higher than 16, the test was terminated, and the patient was given a 20-min rest period before commencement of a new test. Finally, patients were asked to assess Borg RPE for the 4 min at the higher work rate, to ensure that the work rate corresponded to Borg RPE ≈14.

The Ekblom-Bak test equations use the difference in HR between the standard and higher work rate, relative to the increase in power output (PO) to calculate V̇O_2_peak. It is also dependent on the sex and age of the subject, as well as the absolute HR at the standard and higher work rate. HR was registered every 15 s during the last minute of each work rate (3:15, 3:30, 3:45, and 4:00), and the calculated average of these recordings was used as the mean HR for the standard and higher work rate. The calculations of estimated V̇O_2_peak for the Ekblom-Bak test were performed by use of an Excel sheet (Microsoft Corp, Redmond, WA, USA) provided by the Swedish School of Sport and Health Sciences, which included the formula for the latest update of the gender specific equations (30). For women, the equation used was: *V̇O*_2_*peak = 1.84390 − 0.00673 (age) − 0.62578 (ΔHR/ΔPO) + 0.00175 (ΔPO) − 0.00471 (HR at standard work rate)*, and for men the equation was: *V̇O*_2_*peak = 2.04900 − 0.00858 (age) − 0.90742 (ΔHR/ΔPO) + 0.00178 (ΔPO) − 0.00290 (HR at standard work rate)* (24).

### Statistical analysis

All statistical analyses were performed with SPSS statistical software version 27.0 (IBM Corp, Armonk, NY, USA). Descriptive data were controlled for normal distribution by use of normality plots and the Shapiro–Wilk test. All parameters were normally distributed and are presented as mean ± standard deviation (SD), unless otherwise mentioned. To establish the correlation between the 2 tests, Pearson’s correlation coefficient (r) was calculated between the estimated V̇O_2_peak from the Ekblom-Bak test and the directly measured V̇O_2_peak from the CPET. Pearson’s r was classified as weak (< 0.30), moderate (0.30–0.49), or strong (> 0.50). Standard error of estimate was derived from a linear regression model to show the variation around the regression line. The variation in relation to its mean for the difference between estimated and measured V̇O_2_peak was determined by the coefficient of variation. The coefficient of variation was calculated by dividing the SD of the difference between estimated and measured V̇O_2_peak by the mean of the measured V̇O_2_peak. Bland–Altman plot analysis with limits of agreement (LoAs) was performed to determine the bias between the Ekblom-Bak test and the CPET. The bias was determined by calculating the mean difference between estimated and measured V̇O_2_peak, and a 1-sample t-test was used to detect whether the 2 tests were significantly different from each other. LoAs were calculated with the equation: mean difference between estimated and measured V̇O_2_peak ± 1.96 multiplied by the SD of difference between estimated and measured V̇O_2_peak. The LoAs are expected to include 95% of the differences between the tests. Analysis was performed for all patients who completed both tests, and for subgroups based on the patients’ betablocker medication status. Two-tailed significance level was set at *p* < 0.05 for all statistical analysis.

## RESULTS

**Fig. 1:**
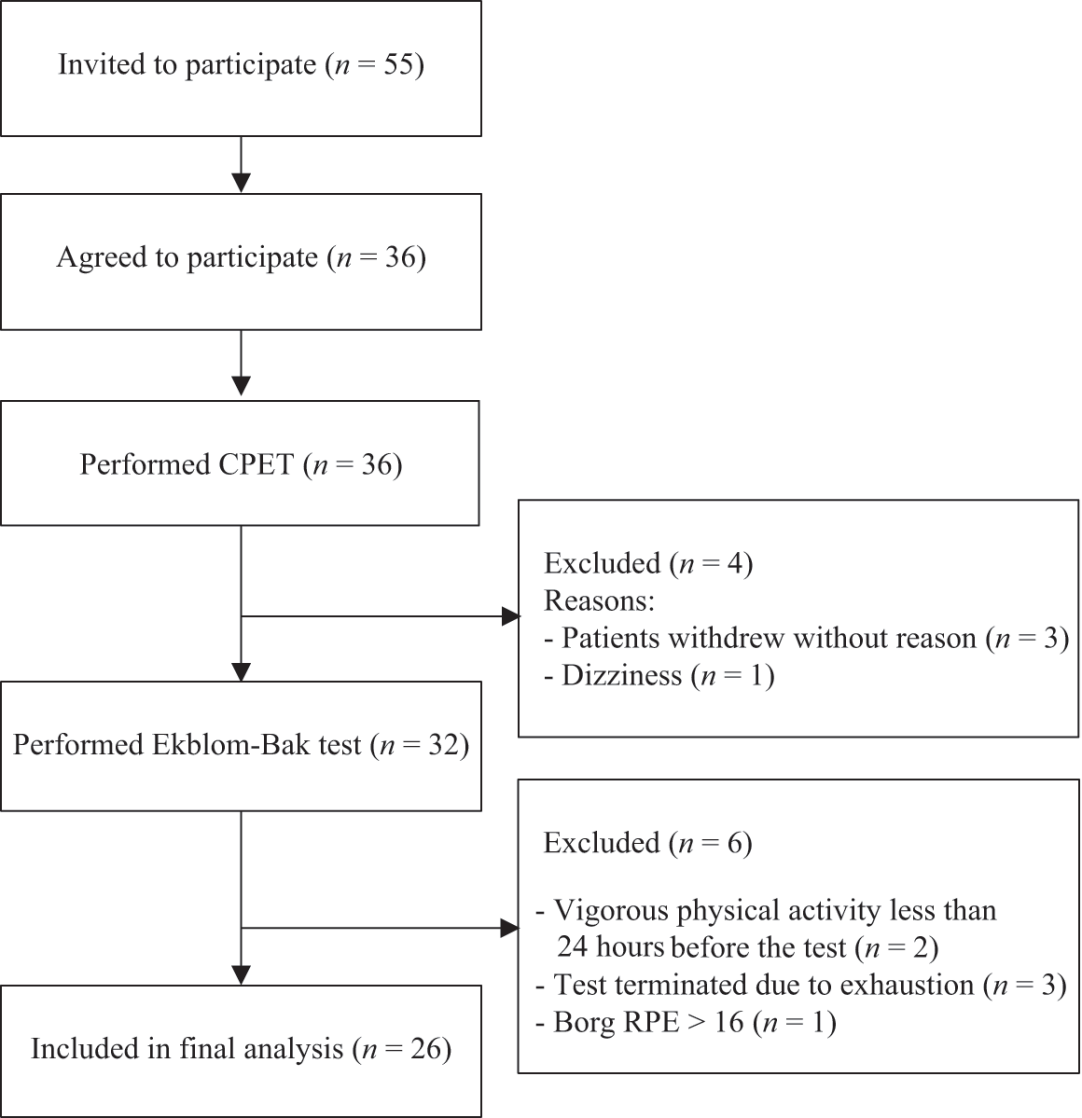
Flowchart describing the recruitment and exclusion process. CPET: cardiopulmonary exercise test; RPE: rating of perceived exertion; W: watt; *n*: number of patients.

The process of recruitment and exclusion is presented in Fig. 1. The final study sample consisted of 26 patients, including 7 women and 19 men with mean age 62 years and mean BMI 29.4. Complete characteristics of the study sample are presented in Table I. The higher work rate for the Ekblom-Bak test ranged from 60–150W, and mean Borg RPE for the higher work rate was 14. No adverse effects occurred during the Ekblom-Bak test.

**Table I:** Characteristics of the study sample

Factor	Total (*n* = 26) Mean (SD)	Using betablockers (*n* = 10) Mean (SD)	Not using betablockers (*n* = 16) Mean (SD)
Age (years)	62 (10)	63 (10)	61 (10)
Height (cm)	175 (9)	176 (9)	175 (9)
Weight (kg)	90.9 (15.6)	86.9 (15.5)	93.4 (15.6)
BMI (kg/m^2^)	29.4 (4.9)	28.2 (6.3)	30.1 (3.7)
HRmax (beats/min)	150 (20)	139 (21.2)	157 (17)
Resting systolic BP (mmHg)	137 (18)	128 (16)	143 (16)
Resting diastolic BP (mmHg)	86 (11)	83 (10)	87 (12)

SD: standard deviation; HRmax: maximal heart rate; BP: blood pressure; *n*: number of patients; BMI: body mass index.

**Fig. 2:**
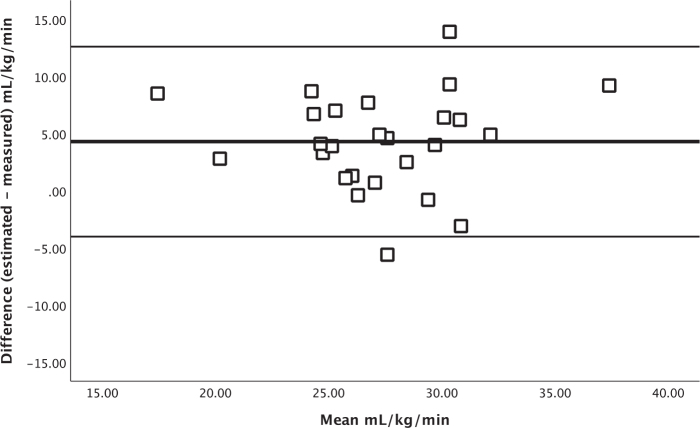
Bland Altman plot, including limits of agreement for estimated and measured V̇O_2_peak. Thick line = mean difference; Thin line = upper and lower limits of agreement.

**Fig. 3:**
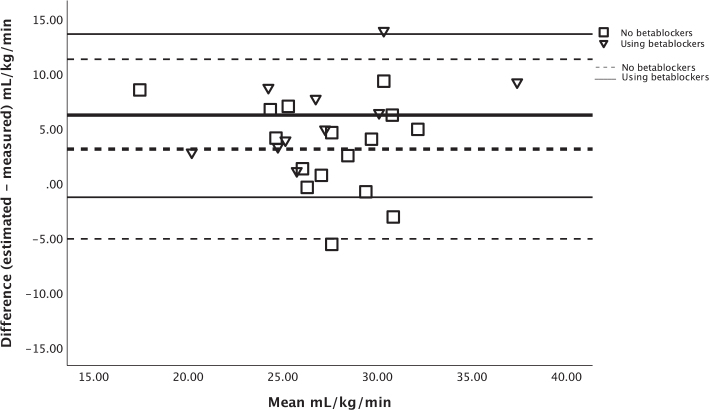
Bland Altman plot, including limits of agreement for estimated and measured V̇O_2_peak. Thick line = mean difference; Thin line = upper and lower limits of agreement.

Fig. 2 shows the agreement between estimated V̇O_2_peak from the Ekblom-Bak test and measured V̇O_2_peak from the CPET in a Bland–Altman plot. The agreement was: bias = 4.3 mL/kg/min (LoA: –4.0 to 12.6 mL/kg/min). For patients using betablockers, agreement of V̇O_2_peak was: bias = 6.2 mL/kg/min (LoA: –1.3 to 13.6 mL/kg/min), while for the patients not using betablockers it was: bias = 3.1 mL/kg/min (LoA: –5.1 to 11.3 mL/kg/min), as illustrated in Fig. 3.

Relevant variables from the Ekblom-Bak test and CPET are presented in Table II. Estimated V̇O_2_peak had a strong correlation to measured V̇O_2_peak in the total study sample and for those patients using betablockers, while there was a moderate correlation for the patients not using betablockers. Pearson’s R was statistically significant across all groups (*p* < 0.05).

**Table II:** Results from the Ekblom-Bak test and the maximal cardiopulmonary exercise test

Factor	Total (*n* = 26)	Using betablockers (*n* = 10)	Not using betablockers (*n* = 16)
*CPET*			
V̇O_2_peak (L/min), mean (SD)	2.29 (0.56)	2.12 (0.60)	2.39 (0.53)
V̇O_2_peak (mL/kg/min), mean (SD)	25.2 (4.3)	24.1 (3.9)	25.8 (4.6)
Respiratory exchange ratio (RER), mean (SD)	1.09 (0.09)	1.09 (0.08)	1.09 (0.10)
*Ekblom-Bak test*			
V̇O_2_peak (L/min), mean (SD)	2.69 (0.57)	2.66 (0.74)	2.70 (0.46)
V̇O_2_peak (mL/kg/min), mean (SD)	29.5 (4.6)	30.3 (5.9)	28.9 (3.6)
Correlation coefficient			
V̇O_2_peak (L/min), *r*	0.769	0.910	0.670
V̇O_2_peak (mL/kg/min), *r*	0.544	0.777	0.496
*Coefficient of variation*			
V̇O_2_peak (L/min), %	17.0	14.8	17.1
V̇O_2_peak (mL/kg/min), %	16.9	15.8	16.3
*SEE*			
V̇O_2_peak (L/min)	0.37	0.27	0.40
V̇O_2_peak (mL/kg/min)	3.7	2.6	4.1

V̇O_2_peak = peak oxygen consumption; SD = standard deviation; SEE = standard error of estimate.

## DISCUSSION

The findings suggest that the Ekblom-Bak test overestimates V̇O_2_peak in patients with CVD. Results also indicate that the Ekblom-Bak test is less accurate in patients using betablockers. Additionally, 3 patients were not able to complete the Ekblom-Bak test due to exhaustion. While the present study reported an overestimation of V̇O_2_peak by 0.40 L/min and 4.3 mL/kg/min, previous validation studies of the Ekblom-Bak test have shown more accurate estimations in healthy individuals. In the study where Björkman et al. (24) updated the equations for maximal O_2_ consumption (V̇O_2_max) estimation, they found the Ekblom-Bak test to underestimate V̇O_2_max by only –0.01 L/min in both men and women. They concluded that their new equation was valid for estimating V̇O_2_max in healthy individuals aged 20–86 years, with CRF levels of 19–76 mL/kg/min. The updated equation has been used in studies conducted later than 2016, as it has replaced the original version created by Ekblom-Bak et al. (25). Validation in an elderly population aged 65–75 years by Väisänen et al. (26), found accurate estimations of V̇O_2_max from the Ekblom-Bak test, with only minor overestimations of 0.02 L/min (*r* = 0.88) and 0.19 mL/kg/min (*r* = 0.77) for absolute and relative V̇O_2_max, respectively. A validation study by Schultz et al. (31), found the Ekblom-Bak test to underestimate V̇O_2_max by 0.48 L/min (*r* = 0.968) and 6.17 mL/kg/min (*r* = 0.980) for absolute and relative V̇O_2_max, respectively. These findings are, however, derived from a relatively small sample size (*n* = 15), with a wide variety in key characteristics, such as age (25–75 years) and activity levels.

To our knowledge, Mijwel et al. (32) have performed the only validation study of the Ekblom-Bak test in a patient population. They investigated the accuracy of V̇O_2_peak estimations in 8 patients with breast cancer undergoing chemotherapy, and found the Ekblom-Bak test to overestimate V̇O_2_peak by 0.79 L/min (*r* = 0.21). These findings along with the results of the present study suggest that the Ekblom-Bak test should be validated in specific patient populations. However, the relatively small sample sizes may introduce some bias in the findings. Nevertheless, due to the relatively wide interval of the LoA and the degree of skewness towards V̇O_2_peak overestimation, the present study indicates the Ekblom-Bak test is not able to accurately estimate V̇O_2_peak in patients with CVD.

The Ekblom-Bak test’s overestimation of V̇O_2_peak in this study may be due to disease-specific characteristics of patients in cardiac rehabilitation. Patients with CVD generally have lower levels of CRF, either because of cardiovascular limitations or because of recent severe events like myocardial infarction or stroke. Most patients in this study had V̇O_2_peak values near the lower part of the V̇O_2_peak range in which the Ekblom-Bak test equation has previously been validated. Individuals with lower V̇O_2_peak may have a higher HR at the standard work rate relative to their maximal HR of the Ekblom-Bak test. This may result in a smaller HR increase between the standard and higher work rate, which would lead to an overestimation of V̇O_2_peak. Also, the Ekblom-Bak test estimates V̇O_2_max, not V̇O_2_peak. This might be a reason why there is an overestimation of V̇O_2_peak in this study, as V̇O_2_peak values may be lower than a person’s true V̇O_2_max. CRF are expressed as V̇O_2_peak in this study because patients with CVD might be inhibited by psychological and physiological factors during their CPET. They may experience fear and lack of motivation, or be restricted by symptoms like unstable angina, arrhythmias, palpitations, dizziness, and dyspnoea. Thus, many patients with CVD would be unable to reach the criteria for a V̇O_2_max measurement, which is normally ≥ 1.1 RER. However, this should not affect the results too much, as every participant included in the final sample had ≥ 1.05 RER.

Three participants were not able to complete the Ekblom-Bak test at the lowest possible higher work rate due to exhaustion. These individuals had V̇O_2_peak ranging from 17.1 mL/kg/min to 22.0 mL/kg/min, compared with an average of 25.2 mL/kg/min for the total sample. This might indicate that for some CVD patients with low CRF the increase from standard to the lowest higher work rate may be too much to cope with. Thus, one might wish to do further research on whether the increments in workload from the standard to the higher work rate could be altered to make the test more suitable for individuals with low CRF.

Many patients with CVD are also prescribed betablockers, which inhibit HR response to exercise and limit maximal HR (33). In fact, analysis in the subgroup using betablockers showed an almost twice as large overestimation of V̇O_2_peak compared with the subgroup not using betablockers. These findings can probably be explained by the inhibitory nature of betablockers, which may cause a limitation in the HR response when workload is increased. Also, Väisänen et al. (26) found that the Ekblom-Bak equation is more likely to overestimate V̇O_2_peak in individuals with low maximal HR. However, these findings are derived from a relatively small sample, so further research on the Ekblom-Bak test’s validity in patients using betablockers should be conducted. Further studies should also investigate the Ekblom-Bak test’s sensitivity to detecting minor changes in CRF as a result of training interventions, as this could be of great interest in a clinical rehabilitation setting.

### Limitations

The present study has some limitations that need to be addressed. First, tests were performed using an electronically braked cycle ergometer rather than the mechanically braked cycle ergometer used to develop the Ekblom-Bak test (30). Using the current electronically braked cycle ergometer causes a slightly lower increase in workload compared with the mechanically braked cycle ergometer according to the test protocol (30W versus 32W). The Ekblom-Bak test has not been validated with an electronically braked cycle ergometer, which might induce minor estimation errors, but the researchers behind the Ekblom-Bak test have made the protocol for electronically braked cycle ergometers available (https://www.gih.se/english/research/laboratories/the-astrand-laboratory/the-ekblom-bak-test). Finally, due to the clinical setting, the 2 tests were integrated into the clinical routines of the cardiac rehabilitation programme. This led to certain necessary considerations in the study design, where the CPET was always scheduled at the start of their rehabilitation stay. Nevertheless, to ensure the strain of performing a CPET would not influence their HR response during the Ekblom-Bak test, patients were given minimum 24 h rest between the tests, in line with the Ekblom-Bak test procedures. In addition, most of the patients in the present study were males, which also could have hampered the generalizability of the results.

### Conclusion

Even though the Ekblom-Bak test seems to be safe in a rehabilitation setting, it overestimated V̇O_2_peak to such an extent that it cannot accurately assess CRF for patients with CVD. Thus, the CPET remains the test of choice when clinically feasible.

## Data Availability

All collected data were filed in a password-protected USB drive, currently stored at Unicare Røros. The datasets generated during and/or analysed during the current study are available from the corresponding author on reasonable request.
